# Sex Hormones and Processing of Facial Expressions of Emotion: A Systematic Literature Review

**DOI:** 10.3389/fpsyg.2018.00529

**Published:** 2018-04-11

**Authors:** Flávia L. Osório, Juliana M. de Paula Cassis, João P. Machado de Sousa, Omero Poli-Neto, Rocio Martín-Santos

**Affiliations:** ^1^Department of Neuroscience and Behavior, Ribeirão Preto Medical School, University of São Paulo, Ribeirão Preto, Brazil; ^2^National Institute of Science and Technology, Translational Medicine (INCT-TM), National Council for Scientific and Technological Development (CNPq), São Paulo, Brazil; ^3^Department of Gynecology and Obstetrics, Ribeirão Preto Medical School, University of São Paulo, Ribeirão Preto, Brazil; ^4^Department of Psychiatry, Institute of Neurosciences, Universidad Autónoma de Barcelona, Barcelona, Spain

**Keywords:** sex hormones, emotion, facial expression, oral contraceptives, estrogen, progesterone, testosterone

## Abstract

**Background:** We systematically reviewed the literature to determine the influence of sex hormones on facial emotion processing (FEP) in healthy women at different phases of life.

**Methods:** Searches were performed in *PubMed, Web of Science, PsycINFO, LILACS*, and *SciELO*. Twenty-seven articles were included in the review and allocated into five different categories according to their objectives and sample characteristics (menstrual cycle, oral contraceptives, pregnancy/postpartum, testosterone, and progesterone).

**Results:** Despite the limited number of studies in some categories and the existence of inconsistencies in the results of interest, the findings of the review suggest that FEP may be enhanced during the follicular phase. Studies with women taking oral contraceptives showed reduced recognition accuracy and decreased responsiveness of different brain structures during FEP tasks. Studies with pregnant women and women in the postpartum showed that hormonal changes are associated with alterations in FEP and in brain functioning that could indicate the existence of a hypervigilant state in new and future mothers. Exogenous administration of testosterone enhanced the recognition of threatening facial expressions and the activation of brain structures involved in the processing of emotional stimuli.

**Conclusions:** We conclude that sex hormones affect FEP in women, which may have an impact in adaptive processes of the species and in the onset of mood symptoms associated with the premenstrual syndrome.

## Introduction

Behavioral research has strengthened the view that sex hormones are involved not only in reproductive behavior or sexual dimorphism, but play an important role in different cognitive and emotional processes, in non-verbal behavior and in the functioning of a number of brain structures (Maki et al., 2002; van Wingen et al., 2011; Poromaa and Gingnell, 2014). Sex hormones act in the central nervous system by modulating the synthesis, release, and metabolism of different neurotransmitters (noradrenaline, dopamine, serotonin, glutamate, and GABA) and neuropeptides and influencing the excitability, synaptic function, and morphological characteristics of neurons (Rosa e Silva and Sá, 2006).

In women, the influence of sex hormones raises special interest because of physiological fluctuations that occur at the different phases of the menstrual cycle, during pregnancy (Klink et al., 2002) and in the postpartum (Bloch et al., 2000). During the normal menstrual cycle, for example, follicle-stimulating *hormone* (*FSH*) and luteinizing *hormone* (*LH*) peaks increase slowly and progressively in both amplitude and frequency shortly after menstrual bleeding. This leads to endometrial thickening and maturation of the ovarian follicle. There is a gradual increase in the production of estradiol until the occurrence of a peak shortly before ovulation. This increase in estradiol induces a significant rise of LH and FSH levels that deflates ovulation, initiating a significant synthesis of progesterone by the corpus luteum until its involution.

Women are also subject to hormone fluctuations associated with the use of oral contraceptives. Most of these drugs inhibit the natural production of ovarian hormones, thus eliminating fluctuations during the menstrual cycle (Fleischman et al., 2010; De Bondt et al., 2013). Hormonal contraceptives, and especially progestagen, simulate a second sustained phase that prevents new peaks of FSH and LH, inhibiting ovulation and promoting their contraceptive effect.

Research has described fluctuations in the levels of estrogen and progesterone and increased vulnerability to mood disorders in women (van Wingen et al., 2011). Also, there is evidence of positive correlations between the concentration of testosterone and antisocial behavior, aggressiveness, and domination behavior in both men and women (Archer, 1991; Book et al., 2001; van Wingen et al., 2011). In addition, investigations have shown alterations in mood and cognitive performance in women taking oral contraceptives (Mordecai et al., 2008; Griksiene and Ruksenas, 2011; Poromaa and Segebladh, 2012).

A non-systematic review on the activation of brain areas involved in emotional regulation associated with sex hormones showed that the amygdala and the medial prefrontal and orbitofrontal cortices are implicated in emotional processes (van Wingen et al., 2011). Toffoletto et al. (2014) have also described the involvement of the insula and the ventral part of the anterior cingulate in this process. All these regions are involved mainly with emotional processing, detection of threat signs, fight or flight response, and regulation of affective states (Toffoletto et al., 2014).

Other investigations about the impact of sex hormones in cognitive and emotional processes showed that these hormones are implicated in visual processing and in facial emotion recognition, since alterations in such abilities were found to be associated with hormone fluctuations over the different phases of the menstrual cycle (Farage et al., 2008; Little, 2013; Poromaa and Gingnell, 2014; Toffoletto et al., 2014).

Considering that facial emotion processing (FEP) is an important element of social cognition that contributes widely to the success of social interactions (Almada, 2012) and that alterations in the processing and recognition of emotional states in others are connected with many psychiatric disorders, the objective of this study was to investigate, through a systematic review of the literature, the influence of endogenous and exogenous sex hormones in the processing of basic facial expressions of emotion in healthy women at different phases of life.

The present study adds to the current literature on the subject as it was aimed at reviewing studies that assessed FEP directly through computerized tasks and used not only the menstrual cycle as a model of the influence of sex hormones, but assessed also women during pregnancy and in the postpartum, in addition to studies that involved the exogenous administration of hormones, including users of oral contraceptives.

## Methods

**Figure 1:**
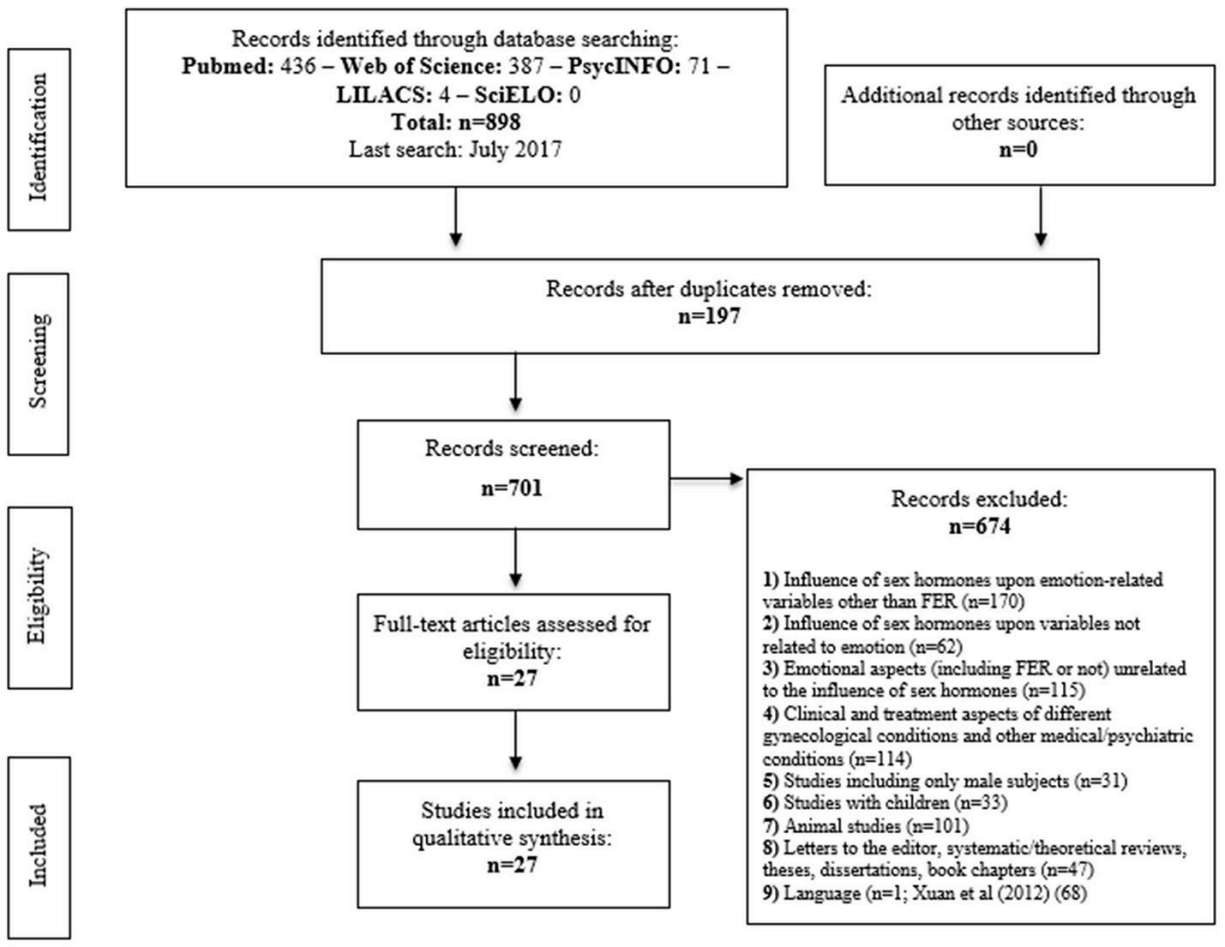
Flow diagram with details on the process of article search and selection for the systematic review.

We performed a systematic search with no time limits (last search in July, 2017) in the electronic databases *Pubmed, Web of Science, PsycINFO, LILACS, and SciELO* using the following MeSH terms: (emotional OR emotion) AND (processing OR recognition OR perception) AND (menstrual cycle OR progesterone OR estrogen OR testosterone OR androgen OR oral contraceptives). We followed the guidelines of the *Preferred Reporting Items for Systematic Reviews and Meta-Analyses* (PRISMA) statement (Moher et al., 2009). The criteria for articles to be included in the review were the following: studies involving healthy women with no age limits, published in Portuguese, English, Spanish, French, and Italian, and which assessed the influence of sex hormones (endogenous and exogenous) on FEP. The exclusion criteria as well as the complete process of article search and selection are shown in Figure 1.

## Results

### General aspects

The searches returned a total of 898 matches for the search terms used. From these, 27 were included in the review after consensus between two of the investigators. The articles were allocated into five different groups according to their objectives and sample characteristics, as follows:

Group 1—Menstrual cycle: observational cross-sectional (group comparisons) and longitudinal studies involving women at different phases of the natural menstrual cycle with the objective of investigating endogenous hormone variations (*n* = 11 studies–19-29).

Group 2—Oral contraceptives: studies with women using oral contraceptives, taking women at different phases of the natural menstrual cycle as a reference, with both observational (*n* = 5) (Maner and Miller, 2014; Hamstra et al., 2015, 2016, 2017; Radke and Derntl, 2016) and experimental (*n* = 2) (Gingnell et al., 2013; Hamstra et al., 2014) designs.

Group 3—Pregnancy/Postpartum: longitudinal studies with women during pregnancy (*n* = 2) (Pearson et al., 2009; Roos et al., 2011) and the postpartum period (*n* = 1) (Gingnell et al., 2015).

Group 4—Testosterone: observational and experimental studies with women aimed at investigating the effects of endogenous (*n* = 1) (Stanton et al., 2009) and exogenous testosterone (single dose between 0.5–0.9 mg; *n* = 4) (van Honk and Schutter, 2007; Hermans et al., 2008; van Wingen et al., 2009; Bos et al., 2013).

Group 5—Progesterone: experimental study with women to assess the acute effects of progesterone through a clinical trial (*n* = 1) (van Wingen et al., 2008).

Of note, we found no studies with samples of healthy women in puberty or in the menopause using the search procedures described above.

### Socio-demographic and methodological aspects

The main socio-demographic and methodological characteristics of the articles included in the review are shown in Table 1.

**Table 1:** Socio-demographic and methodological characteristics of the articles included in the review.

**Author (Year)**	**Country**	**Design/sample**	**N**	**Age mean (SD)**	**Level of education (years) mean (SD)**	Cycle phase/pregnancy^*^	**Method of hormone analysis**	**Hormone levels (units) mean (SD)**
**MENSTRUAL CYCLE**								
Pearson and Lewis, 2005	ENG	Cross-sectional Group comparison Correlational University students	12 11 13 14	20.0 (18–22)	NI	EFP (menses: 1–2 d) EFP/LFP (pre-ovulation: 3–12 d) ELP (ovulation: 13–17 d) ELP/LLP (after ovulation: 18–28 d)	_	_
Derntl et al., 2008a	AUT	Cross-sectional Group comparison Correlation University Student	15 17	23.7 (2.7) 24.0 (3.8)	16.7 (3.1) 16.7 (3.2)	EFP/LFP (NI) ELP/LLP (NI)	ES/P (blood)^a^	**ES (pg/ml)/P (ng/ml)** 39.7 (14.1)/0.66 (0.2) 135.7 (93.3)/4.2 (5.1) *-Level E: EFP/LFP < ELP/LLP* *-Level P: EFP/LFP < ELP/LLP*
Derntl et al., 2008b	AUT	Cross-sectional Group comparison Correlational University Student	11 11	23.4 (3.4) 25.3 (3.9)	16.5 (3.7) 17.6 (3.1)	EFP/LFP (1–14 d) ELP/LLP (15–28 d)	ES/P (blood)^a^	**ES (pg/ml)/P (ng/ml)** 44.8 (16.6)/0.62 (0.3) 135.7 (93.3)/4.3 (4.5) *-Level E: EFP/LFP < ELP/LLP* *-Level P: EFP/LFP < ELP/LLP*
Guapo et al., 2009	BRA	Cross-sectional Group comparison Correlational Students	11 9 10	22.36 (3.07) 21.11 (3.29) 22.70 (2.45)	NI	EFP (1–5 d) LFP (12–14 d) ELP/LLP (21–23 d)	E/P (blood)^b^	**E/P (NI)** 43.85 (26.63)/0.55 (0.30) 126.59 (86.62)/0.73 (1.13) 110.54 (34.58)/9.23 (4.60) *-Level E: EFP < LFP = ELP/LLP* *-Level P: EFP = LFP < ELP/LLP*
Derntl et al., 2013	AUT	Cross-sectional Group comparison University students	20 17	25.1 (3.3) 26.0 (3.3)	18.3 (2.2) 18.4 (1.7)	EFP (2–5 d) ELP/LLP (18–25 d)	ES/P (saliva)^c^	**ES/P (pg/ml)** 2.6 (1.3)/61.1 (17.9) 3.7 (1.2)/221.5 (109.9) *-Level E: EFP < ELP/LLP* *-Level P: EFP < ELP/LLP*
Kamboj et al., 2015	ENG	Cross-sectional Correlational Community	16 14 14	22.50 (3.37) 23.54 (4.26) 23.13 (3.58)	16.00 (1.92) 15.69 (2.43) 15.73 (2.12)	LFP (7–13 d) ELP (15–19 d) LLP (22–27 d)	ES/P (saliva)^d^	**ES/P (pg/ml)** 5.71 (1.98)/72.61 (47.72) 6.11 (2.57)/125.95 (124.24) 5.79 (2.34)/211.86 (181.36) *-Level E: LFP = ELP = LLP* *-Level P: LFP < ELP/LLP*
Conway et al., 2007	UK	Longitudinal University students	22	19.30 (1.63)	>12	NI	P (saliva)	NI
Rubinow et al., 2007	USA	Longitudinal Community	27	33.5 (7.0)	NI	EFP/LFP (5–11 d) ELP/LLP (19–28 d)	_	_
Rubin et al., 2011	USA	Longitudinal Community	31	27.55 (6.67)	NI	EFP (2–4 d) ELP/LLP (20–22 d)	E/P (blood)^e^	NI
Gingnell et al., 2012	SWE	Longitudinal Community	15	33.7 (8.4)	NI	EFP/LFP (7–10 d) LLP (23 d)	ES/P (NI)^f^	**ES (pmol/l)/P (nmol/l)** 303 (150)/3.4 (3.8) 414 (211)/21.8 (13.1) *-Level E: EFP/LFP < LLP* *-Level P: EFP/LFP < LLP*
Zhang et al., 2013	CHN	Longitudinal Community	26	20.6 (1.5)	NI	EFP (1–4 d) LFP/ELP (11–18 d) LLP (23–29 d)	_	_
**ORAL CONTRACEPTIVES**								
Maner and Miller, 2014	USA	Cross-sectional Group comparison Correlational Community	22 21	19.4 (2.0)	NI	NC (*n* = 10:16–28 d) (*n* = 3: 13–15 d) (*n* = 3: 1–12d) OC	P	**NC**: Standartized Estimation (Garver-Apgar et al., 2008) **OC**: NI
Hamstra et al., 2015	NLD	Cross-sectional Group comparison University students	40 44	20.2 (0.4) 20.4 (0.3)	NI	NC (*n* = 11:6–14 d) (*n* = 2 9: 15–26) OC	–	**OC** = 91% with estrogen and/or progestin as compounds Time of use: 86% > 1 year
Radke and Derntl, 2016	GER	Cross-sectional Group comparison Community	18 25 30	23.9 (1.8) 22.7 (1.3) 22.9 (2.3)	NI	NC (NI) OFFP OC	–	**OC** = 100% monophasic combined Time of use: > 3 months
Hamstra et al., 2016	NLD	Cross-sectional Group comparison Students	21 23 49	20.4 21.0 20.4	NI	NC (2–5 d) NC (18–25 d) OC/OFFP	–	**OC** = ethinylestradiol (0.03 mg) = levenorgestrel (0.15mg)
Hamstra et al., 2017	NLD	Cross-sectional Group comparison Correlational NI	39 57	20.9 (0.3) 20.7 (0.2)	NI	NC (2–6 d) (18–25d) OC OFFP	ES/P (saliva) ^g^	**ES/P (pg/ml)** 2.6 (0.2)/72.9 (17.6) 3.4 (0.2)/148.2 (16.1) 2.1 (0.2)/53.7 (5.7) 2.3 (0.2)/43.6 (2.9)
Gingnell et al., 2013	SWE	RCT, Placebo Community	17 17	24.5 (3.3) 25.5 (5.0)	15.6 (1.6) 15.0 (2.3)	NC (1-10/15-21) OC	OC = ethinyl estradiol (30 μg) levonorgestrel (0.15mg)/21 days or placebo	NI
Hamstra et al., 2014	NLD	RCT, Placebo University students	14 26	22.2 (0.7) 20.6 (0.5)	NI	NC (NA) OC	–	**OC** = NI
**PREGNANCY/POSTPARTUM**								
Pearson et al., 2009	ENG	Longitudinal Community of midwives	76	29.5 (18–41)	< 14 (43%) > 14 (57%)	EPG (7-14 wk) LPG (33-39 wk)	_	_
Roos et al. (2011)	SA	Longitudinal Group comparison Community of midwives	10 12 10 9	24.8 (5.6) 25.3 (5.7)	11.5 (1.2) 11.8 (0.8)	1st trimester (13-14 wk) 2nd trimester (22-23 wk) 3rd trimester (32-33 wk) Non-pregnancy (NI)	E/P/T (blood/saliva)	**E (pmol/l)/P (nmol/l)/T (pmol/l)** 18767.7(10364.8)/ 128.8(45.8)/11.7 (5.1) 53540.0(42303.6)/ 199.5(77.0)/8.8 (44.3) 10224.2(87462.9)/ 27.5(141.4)/9.0 (6.3) NA/NA/3.5 – 39.0
Gingnell et al., 2015	SWE	Longitudinal Group comparison General Hospital/Community	13 15	32.8 (4.2) 33.7 (8.4)	12 (92.3%) 13 (86.7%)	EPP (2d) LPP (4-6 wk) EFP/LFP (NI) ELP/LLP (NI)	ES/P (NI)^h^	**ES (pmol/l)/P (nmol/l)** 1590 (709)/45.6 (37.7) 120 (56)/0.8 (0.5) 304 (150)/3.4 (3.8) 414 (211)/21.8 (13.1) *-Level E: EPP > ELP/LLP* *EPP > EFP/LFP, ELP/LLP* *LPP < EFP/LFP, ELP/LLP* *-Level P: EPP > LPP* *EPP > EFP/LFP, ELP/LLP* *LPP < EFP/LFP, ELP/LLP*
**TESTOSTERONE**								
Stanton et al., 2009	USA	Cross-sectional Correlational NI	14	20.96 (2.18)	NI	NA	T (saliva)^i^	**T (pmol/l)** 17.28 (1.52)
van Honk and Schutter, 2007	NLD	RCT, Placebo Cross-over NI	16	19-26	NI	NC: EFP (NA)	T (0.5mg) sublingual, single dose or placebo (blood)	NI
Hermans et al., 2008	NLD	RCT, Placebo Cross-over Community	12	22.6 (18–28)	NA	10 OC/2 NC: EFP/LFP (NI)	T (0.5mg) sublingual, single dose or placebo (saliva)	NI
van Wingen et al., 2009	NLD	RCT, Placebo, Cross-over NI	17 25	23 (19–30) 42 (37–50)	NI	NC (1-7 d) PRE-M (1-7 d)	T (0.9 mg) nasal, single dose or placebo (blood)	**T (pmol/l)** 964.8 (58.3) 672.7 (52.7) 3398.1 (404.5)
Bos et al., 2013	NLD	RCT, Placebo, Cross-over University students	12	20.4 (18–25)	NI	9 OC/3 NC: EFP/LFP (NI)	T (0.5mg) sublingual, single dose or placebo (saliva)^j^	NI
**PROGESTERONE**								
van Wingen et al., 2008	NLD	RCT, Placebo, Cross-over NI	18	24 (19–39)	NI	NC: EFP (Bloch et al., 2000; Klink et al., 2002; Rosa e Silva and Sá, 2006; Fleischman et al., 2010; van Wingen et al., 2011; Poromaa and Gingnell, 2014)	P (400 mg) orally or placebo (blood)	**P (nmol/l)** 1.94 (0.29)/16.83 (6.17)

*AUT, Austria; BRA, Brazil; CHN, China; ENG, England; GER, Germany; NLD, Netherlands; SA, South Africa; SWE, Sweden; USA, United States of America; UK, United Kingtom; d, days; E, estrogen; EFP, early follicular phase; ELP, early luteal phase; EPG, early pregnancy; EPP, early postpartum; ES, estradiol; LFP, late follicular phase; LLP, late luteal phase; LPG, late pregnancy; LPP, late pospartum; N, number of subjects; NC, natural menstrual cycle; NI, not informed; OC, oral contraceptive users; OFFP, pill-free week; P, progesterone; PRE-M, premenopausal; RCT, randomized clinical trial; sd, standard deviation; T, testosterone; wk, week*.

a *Electrochemiluminescence immunoassay (ECLIA, Johnson et al., 1993)*.

b *Chemiluminescent immunoassay (Immulite, 2000)*.

c *Enzyme-linked immunoassay method from DRG (DRG Marburg, Germany)*.

d *Luminescence immunoassay kits (Hamburg, Germany)*.

e *Estradiol by double-antibody radioimmunoassay (RIA; Diagnostic Products, Los Angeles, CA), progesterone by Coat-a-Count coated tube RIA (Diagnostic Products, Los Angeles, CA)*.

f *Competitive immunometry electrochemistry luminescence*.

g *Highly sensitive luminescence assays of IBL at Ganzimmun Diagnostics AG (D)*.

h *Competitive immunometric electrochemical luminescence (Cobas e601)*.

i *Solid-phase Coat-A-Count^125^ I radioimmunoassays (Schultheiss et al., 2004)*.

j *Radio–immunoassay employing a polyclonal antitestosterone-antibody X placebo (Dr. Pratt AZG 3290)*.

* *The phases of the menstrual cycle/pregnancy were categorized according to the parameters described by Poromaa and Gingnell (2014) considering the days of the menstrual cycle when women were evaluated*.

The samples in the articles reviewed had a median of 32 participants, with a mean age of 25 years. In general, the women were recruited in university (*n* = 9) and community (*n* = 8) settings.

In the menstrual cycle group, 73% (*n* = 8) of the studies measured hormone concentrations using standardized techniques, with samples collected from blood and saliva. Among the studies included in the oral contraceptives groups, most (*n* = 4; 60%) presented some information regarding the hormonal components of the oral contraceptives used, which has been described as a positive methodological factor (Poromaa and Gingnell, 2014).

In respect to the procedures of the FEP tasks, 18 studies used static stimuli and 7 used dynamic stimuli, regarded as having greater ecological validity (Torro-Alves, 2013; Torro-Alves et al., 2016). The most commonly used stimuli set (15 studies) was the series Pictures of Facial Affect (Ekman and Friesen, 1976). Most of the studies (*n* = 12) assessed at least five emotions, displayed by actors of both sexes. The minimum number of stimuli used in the studies was 16 and the maximum was 240, with a median of 40. The outcomes investigated were accuracy (*n* = 22), response bias/error pattern (*n* = 3), response time (*n* = 13), intensity of emotion (*n* = 2), and brain activation (*n* = 10). The aspects of the FEP tasks are described in greater detail in Supplementary Table 1.

## Outcomes

The results concerning the outcomes of accuracy, emotional intensity, response bias, and response time are presented in Table 2, while neuroimaging outcomes are shown in Table 3.

**Table 2:** Main results in tasks of facial emotion recognition for the outcomes accuracy, emotional intensity, response time, and response bias.

**Study**	**Design**	**Results**
**MENSTRUAL CYCLE**		
Pearson and Lewis, 2005	Cross-sectional Group comparison Correlational	↑ accuracy during pre-ovulation/ovulation for fear (LFP >EFP: *p* < 0.05) ↑ E ↑ accuracy for fear (*r* = 0.987; *p* < 0.01) No significant difference for response time
Derntl et al., 2008a	Cross-sectional Group comparison Correlational	↑ accuracy during the early and late follicular phase (EFP/LFP > ELP/LLP: *p* = 0.02) ↑ P ↓ total accuracy (*r* = −0.41; *p* = 0.023) *E* = no correlation with total accuracy No significant emotion-by-group interaction for accuracy (*p* = 0.43) ↑ E ↑ number of stimuli classified as anger (*r* = 0.45; *p* = 0.013) ↑ P ↑ number of stimuli classified as anger (*r* = 0.40; *p* = 0.01) ↑ P ↓ number of stimuli classified as neutral (*r* = −0.56; *p* = 0.002) ELP/LLP: ↑ number of stimuli classified as anger (*p* = 0.011) and disgust (*p* = 0.014)
Derntl et al., 2008b	Cross-sectional Group comparison Correlational	↑ accuracy during the follicular phase (*p* = 0.011) No significant emotion-by-group interaction for accuracy (*p* = 0.62) No significant correlations between hormone levels (E/P) and accuracy No significant association between hormone levels and response times (*p* = 0.74)
Guapo et al., 2009	Cross-sectional Group comparison Correlational	↑ accuracy during the early follicular phase for anger (*p* = 0.004) and sadness (*p* = 0.048) (EFP > LFP, ELP, LLP) No significant association for disgust (*p* = 0.48), fear (*p* > 0.05), happiness (*p* = 0.85) and surprise (*p* = 0.53) ↑ E ↓ accuracy for anger (*r* = −0.38, *p* = 0.04)
Derntl et al., 2013	Cross-sectional Group comparison	↑ accuracy during the early follicular phase (EFP > ELP/LLP – *p* = 0.04, $n_{p}^{2}$ = 0.11) No difference between groups for response times (*p* = 0.32) No significant emotion-by-group interaction for accuracy (*p* = 0.16) and response times (*p* = 0.60)
Kamboj et al., 2015	Cross-sectional Correlational	*P* = no significant correlation with accuracy (*r* = −0.26 to 0.05, *p* > 0.05) ↑ E ↓ accuracy for anger (*r* = −0.45, *p* = 0.005) ↑ P ↓ number of stimuli classified as neutral (*r* = −0.32, *p* > 0.05) ↑ E ↓ number of stimuli classified as disgust (*r* = −0.34, *p* > 0.05) ↑*P* ↑ response time for anger (*r* = 0.38, *p* < 0.05), happiness (*r* = 0.40, *p* < 0.005), sadness (*r* = 0.51, *p* < 0.001) and neutral faces (*r* = 0.42, *p* < 0.005) *E* = no correlation with response times (*r* = 0.10–0.28, *p* > 0.05)
Conway et al., 2007	Longitudinal	↑*P* = increased tendency to perceive expressions of fear (*p* = 0.03) and disgust (*p* = 0.06) with averted gaze as more intense than those with direct gaze ↑*P* = no significant association with happiness (*p* = 0.278)
Rubinow et al., 2007	Longitudinal	No difference in response bias between phases (EFP/LFP = ELP/LLP)
Rubin et al., 2011	Longitudinal	↑ accuracy during the early follicular phase (EFP > ELP/LLP; *p* < 0.05, *d* = 1.00) No significant emotion-by-group interaction for accuracy No significant correlation between hormone levels (E/P) and accuracy No difference in response times between phases (*p* > 0.05)
Gingnell et al., 2012	Longitudinal	No difference in accuracy (*p* = 0.29, *d* = 0.40) and response times (*p* = 0.57, *d* = 0.21) between phases
Zhang et al., 2013	Longitudinal	No difference in accuracy (*p* = 0.52) LLP × EFP: *d* = 0.0 LLP × LFP/ELP: *d* = 0.06 EFP × LFP/ELP: *d* = 0.06
**ORAL CONTRACEPTIVES**		
Maner and Miller, 2014	Cross-sectional Group comparison Correlational	OC: ↓ accuracy for anger, disgust, fear, sadness (OC < NC; *p* = 0.037, $n_{p}^{2}$ = 0.20) NC: ↑ P ↑ accuracy for anger, disgust, fear, sadness (*r* = 0.45; *p* = 0.037) OC = P no significant correlation with accuracy (*r* = 0.10, *p* = 0.67)
Hamstra et al., 2015	Cross-sectional Group comparison	OC = ↓ accuracy for anger (OC < N; *p* = 0.02, *n*^2^*_*p*_* = 0.064) and sadness (*p* = 0.01, *n*^2^*_*p*_* = 0.07) OC = ↑ intensity for the recognition of anger (*p* = 0.001; $n_{p}^{2}$ = 0.33) and fear (*p* = 0.006, $n_{p}^{2}$ = 0.26) (OC > NC)
Radke and Derntl, 2016	Cross-sectional Group comparison	No difference in accuracy between groups (*p* > 0.54) No significant emotion-by-group interaction for accuracy (*p* > 0.54)
Hamstra et al., 2016	Cross-sectional Group comparison	OC with MR HT 1/3: ↓ accuracy (OC MR HT1/3 < NC [18–25 days] MR HT 1/3; *p* = 0.01; $n_{p}^{2}$ = 0.23) No difference between OC and NC (2–5 days)
Hamstra et al., 2017	Cross-sectional Group comparison Correlational	OC: ↓ accuracy for happiness (OC < NC; *p* = 0.017; hp^2^ = 0.07) and sadness (OC < NC; *p* = 0.048; hp^2^ = 0.05) OC: ↓ response time for anger (OC < NC; *p* = 0.047; hp^2^ = 0.05) and happiness (OC < NC; *p* = 0.005; hp^2^ = 0.009) E ↑sadness (*r* = 0.20; *p* = 0.048) ↑ E ↑ happiness for MR HT 1/3 (*r* = 0.39; *p* = 0.008)
Gingnell et al., 2013	RCT, Placebo	No differences in accuracy and response time between groups or phases of the menstrual cycle
Hamstra et al., 2014	RCT, Placebo	OC = ↓ accuracy for anger, (*p* = 0.001, $n_{p}^{2}$ = 0.27), sadness (*p* = 0.03, $n_{p}^{2}$ = 0.37) and disgust (*p* = 0.02, $n_{p}^{2}$ = 0.13) (OC < NC) OC = ↓ response time for disgust ($n_{p}^{2}$ = 0.08, *p* = 0.08) and sadness ($n_{p}^{2}$ = 0.14, *p* = 0.02)
**PREGNANCY/POSTPARTUM**		
Pearson et al., 2009	Longitudinal	↑ accuracy during late pregnancy for anger (*p* < 0.05; *d* = 0.23), disgust (*p* < 0.01; *d* = 0.47) and fear (*p* < 0.01; *d* = 0.28) (LP > EP)
Gingnell et al., 2015	Longitudinal Group comparison	No difference in accuracy (EPP × LPP × EFP/LFP × ELP/LLP, *p* = 0.84) and response time (*p* = 0.89) between groups
**TESTOSTERONE**		
van Honk and Schutter, 2007	RCT, Placebo, Cross-over	*T* = no significant overall reduction in accuracy [*T* = PL, Z (1.15) = −1.80, p_rep_ = 0.85, *r* = 0.33] T = ↓ accuracy for threatening faces [threat > non-threat – Z (1.15) = −1.99, p_rep_ = 0.88, *r* = 0.36] T = no significant results for disgust [Z (1.15) = −1.12] and fear [Z (1.15) = −1.28] T = ↓ accuracy for anger [Z (1.15) = −2.28, p_rep_ = 0.92, *r* = 0.42] No difference in emotional intensity between groups (*T* = PC; *p* > 0.25)
van Wingen et al., 2009	RCT, Placebo, Cross-over	*T* = no association with accuracy (*p* > 0.4) or response time (*p* > 0.70)
**PROGESTERONE**		
van Wingen et al., 2008	RCT, Placebo, Cross-over	*P* = no association with accuracy (*p* > 0.05) or response time (*p* > 0.05)

*d, Cohen's d; E, estradiol; EFP, early follicular phase; ELP, early luteal phase; EP, early pregnancy; EPP, early postpartum; LFP, late follicular phase; LLP, late luteal phase; LP, late pregnancy; LPP, late postpartum; ${\text{n}}_{\text{p}}^{2}$, partial eta^2^; MR HT 1/3, mineralocorticoid receptor haplotype 1/3 carriers; NC, naturally cycling women; OC, oral contraceptive users; P, progesterone; p, p-value; RCT, randomized clinical trial; r, correlation coefficient; T, testosterone; ↑, increase; ↓, decrease*.

**Table 3:** Main neuroimaging outcomes in facial emotion recognition studies.

**Author**	**Design**	**Results**
**MENSTRUAL CYCLE**		
Derntl et al., 2008b	Cross-sectional Group comparison Correlational	**Functional data** •Bilateral amygdala activation to all emotions and neutral faces in the follicular phase **ROI analysis** •Stronger amygdala response in EFP/LFP (*p* = 0.049) •Follicular phase = stronger amygdala activation, bilaterally for disgust (EFP/LFP > ELP/LLP: left, *p* = 0.038; right, *p* = 0.001) and right-sided for happiness (EFP/LFP > ELP/LLP: *p* = 0.037) •↓ P ↑ amygdala response to fear (r = −0.440, *p* = 0.046), neutral faces (*r* = −0.488, *p* = 0.025) and sadness (*r* = −0.442, *p* = 0.045) •E = no significant correlation **Whole-slab analysis** •EFP/LFP—stronger response of medial temporal regions during recognition of disgust (*p* = 0.002) and sadness (*p* = 0.001) •EFP/LFP—stronger activation in the hippocampal area for neutral faces (*p* = 0.001)
Gingnell et al., 2012	Longitudinal Correlational	**Reactivity** •↑ left amygdala reactivity in the late luteal phase (LLP > EFP/LFP) **Correlation** •P = no correlation with amygdala reactivity •E = no correlation with amygdala reactivity **Habituation of bilateral amygdala reactivity** •EFP/LFP = right amygdala first session > second session (*p* = 0.02); left amygdala second session > first session (*p* = 0.003) •LLP = no significant clusters found
**ORAL CONTRACEPTIVES**		
Gingnell et al., 2013	RCT, Placebo	**Reactivity** •OC ↓ left insula reactivity •OC ↓ reactivity in left middle frontal gyrus and bilateral inferior frontal gyrus (OC < NC, *p* < 0.001) •OC ↓ reactivity in the bilateral inferior frontal gyri (pretreatment > after treatment, *p* < 0.001)
**PREGNANCY/POSTPARTUM**		
Roos et al. (2011)	Longitudinal	**Activation** •↑ prefrontal cortex activation to fearful faces compared to rest in both pregnancy and non-pregnancy •2nd trimester: ↑ prefrontal cortex activation to fearful faces (2nd trimester > 1st and 3rd trimesters) •↑ prefrontal cortex activation significantly associated with increased levels of testosterone in pregnancy
Gingnell et al., 2015	Longitudinal Group comparison	**Reactivity** •EPP ↓ reactivity in the right insula, bilateral inferior frontal gyrus, and left middle frontal gyrus (EPP < LPP, *p* < 0.005) •Increased reactivity in the insula and inferior frontal gyrus in postpartum compared to non-pregnant women •No significant clusters found •No correlation between brain reactivity and E/P to any of the postpartum time points (p = NA)
**TESTOSTERONE**		
Stanton et al., 2009	Cross-sectional Correlational	No effect of testosterone levels on amygdala BOLD response to angry-neutral face contrasts (left amygdala: *r* = 0.14, *p* = 0.63 – right amygdala: *r* = 0.23, *p* = 0.42)
Hermans et al., 2008	RCT, Placebo, Cross-over	•T ↑ left orbitofrontal cortex to angry faces (*p* < 0.001)
van Wingen et al., 2009	RCT, Placebo, Cross-over	**Neural response** •T ↑ left amygdala (*p* < 0.001) and right amygdala (*p* = 0.008) reactivity in middle-aged women •T ↑ neural response to emotional faces in the inferior frontal and middle temporal gyri (*p* < 0.001) •T ↓ neural response to emotional faces in the precuneus (*p* < 0.001) **Correlation** •↑ T ↑ neural responses in the middle frontal gyrus, superior frontal gyrus, supramarginal gyrus, and precuneus (*p* < 0.001) •↑ T ↓ responses in the orbitofrontal cortex and occipital gyrus •T no significant correlations with amygdala reactivity **ROI analysis** •↑ amygdala reactivity in young compared to middle-aged women (left: *t*(40) = 3.1, *p* = 0.004; effect size: *r* = 0.44; right: *t*(40) = 2.1, *p* = 0.043, *r* = 0.31) •T ↑ left amygdala reactivity in middle-aged women (left: *t*(24) = 2.2, *p* = 0.035, *r* = 0.41; right: *t*(24) = 1.3, *p* = 0.19, *r* = 0.27)
Bos et al., 2013	RCT, Placebo, Cross-over	•T↑ right amygdala activation (right panel) for fear and happiness (*T* > placebo; *p* < 0.05) **Amygdala subregions** •SFA: most prominent effect of testosterone (*p* = 0.007, $n_{p}^{2}$ = 0.63) •BLA: trend toward stronger responses after testosterone (*p* = 0.098, $n_{p}^{2}$ = 0.37) •CMA: no effect of drug (*p* = 0.74, $n_{p}^{2}$ = 0.06) •Significant main effect or interaction involving hemispheres was found in neither of the three subregions **BOLD response to presentation of faces across drug conditions** •SFA: significant responses (*p* = 0.001, *n*^2^*_*p*_* = 0.75) •BLA: significant responses (*p* < 0.001, *n*^2^*_*p*_* = 0.84) •CMA: no significant responses (*p* > 0.005) **Correlation** •Testosterone not correlated with amygdala activation (*p* > 0.05)
**PROGESTERONE**		
van Wingen et al., 2008	RCT, Placebo, Cross-over	**Reactivity** •P ↑ response in right (*p* = 0.001) and left amygdala (*p* = 0.005) •*P* = no influence on neural activity in other brain areas

*BLA, basolateral amygdala; CMA, central-medial amygdala; E, estradiol; EFP, early follicular phase; ELP, early luteal phase; EPP, early postpartum; LFP, late follifular phase; LLP, late luteal phase; LPP, late postpartum; ${\text{n}}_{p}^{2}$, partial eta^2^; NC, naturally cycling women; NA, not available; OC, oral contraceptive users; P, progesterone; RCT, randomized clinical trail; r, correlation coefficient; SFA, superficial amygdala; T, testosterone; ↑, increase; ↓, decrease*.

As seen in Table 2, five studies in the menstrual cycle group described an association between the follicular phase (specially the late follicular phase) and the pre-ovulatory/ovulatory phases (higher concentration of estradiol/estrogen and lower concentration of progesterone) and increased accuracy in emotional recognition in general, with variable effect sizes ranging from small to large (Pearson and Lewis, 2005; Derntl et al., 2008a,b, 2013; Rubin et al., 2011). Conversely, two studies reported no such associations (Gingnell et al., 2012; Zhang et al., 2013). However, when considering the size of the differences between groups, the study by Gingnell et al. (2012) pointed to increased accuracy in emotional recognition in the follicular phase (*d* = 0.40). The same occurred in the study by Zhang et al. (2013), however, the effect size in this case was very small (*d* = 0.06). No associations or differences in the recognition of specific emotions were found (Derntl et al., 2008b, 2013; Rubin et al., 2011). Thus, taken together the results suggest an advantage in the global recognition of emotions in the follicular phase.

However, specific analyses about the association between hormone levels (independently of menstrual cycle phase) and accuracy of emotional judgment showed that higher estrogen/estradiol levels were linked to improved recognition of fear (Pearson and Lewis, 2005) and decreased accuracy in the recognition of anger (Guapo et al., 2009; Kamboj et al., 2015) and disgust (Kamboj et al., 2015). In respect to progesterone, the associations found were less evident, but increased progesterone levels have been associated with global impairment in FEP consisting of increased response time, increased response biases, negative biases, and decreased accuracy of emotional judgment (Conway et al., 2007; Derntl et al., 2008a; Kamboj et al., 2015).

Concerning response time, only one study (Kamboj et al., 2015) described an association between higher progesterone levels and increased response time for the recognition of anger, happiness, sadness, and neutral faces.

Neuroimaging studies with women at different phases of the natural menstrual described associations between activation of the amygdala during FEP tasks and hormone levels, although not in the same direction. While Derntl et al. (2008b) found increased activation of the amygdala associated with emotion recognition in the follicular phase, that is, when progesterone levels are reduced, Gingnell et al. (2012) described increased activation of the left amygdala in the luteal phase relative to the follicular phase in healthy controls, in addition to lack of amygdala habituation. Still in the latter investigation, progesterone levels were not associated with changes in the activation of brain structures during the recognition of emotions. It should also be noted that there were no associations between estradiol levels and amygdala responsiveness during the recognition of facial emotions in these two studies. It should be noted that the two studies used different samples, FEP tasks, and neuroimaging protocols, which may explain the discrepancies between their findings.

In the studies grouped under the name of “oral contraceptives,” Gingnell et al. (2013) compared the FEP performance of women in their natural menstrual cycle with a history of negative mood during the previous use of contraceptives and women on contraceptive treatment for 21 days in a placebo-controlled clinical trial. The authors found no difference between the groups concerning the accuracy of emotional recognition; however, they described reduced activation of the insula, left middle frontal gyrus and bilateral inferior frontal gyri in women taking oral contraceptives compared to placebo. These brain regions are involved in the response to positive and saliency emotional stimuli and take part in different social functions such as language and empathy (Gingnell et al., 2013).

While investigating the effects of a corticosteroid (fludrocortisone) in a clinical trial, Hamstra et al. (2014) found that the use of oral contraceptives by women in their sample was associated with lower accuracy in the recognition of sadness, anger, and disgust. Four other cross-sectional studies reached the same results for the same emotions when comparing users of oral contraceptives and women in their natural menstrual cycle (Maner and Miller, 2014; Hamstra et al., 2015, 2016, 2017), although another investigation with the same methodological design did not support these findings (Radke and Derntl, 2016). Hamstra et al. (2017) also described impaired recognition of facial happiness in association with the use of oral contraceptives.

Among the studies that included pregnant women and women in the postpartum period, Pearson et al. (2009) described an enhancement in the recognition of anger, disgust, and fear in the late stages of pregnancy, when the levels of progesterone and estrogen are theoretically higher. The effect size of this finding was medium in comparison with women in the early stages of pregnancy.

In the postpartum period, Gingnell et al. (2015) found no difference in emotional recognition accuracy between women at different phases of the postpartum and the menstrual cycle, suggesting that estradiol and progesterone concentrations do not affect FEP. Neuroimaging data, however, showed reduced activation in the right insula, bilateral inferior frontal gyri, and left medial frontal gyrus in women in the immediate postpartum (reduction in estrogen and progesterone levels) compared to the late postpartum. The activation of the insula and the inferior frontal gyrus was also higher in women in the postpartum compared to non-pregnant subjects.

In regard to the effects of the acute administration of testosterone on FEP, the studies reviewed described reduced accuracy in the recognition of angry and threatening faces following the oral administration of 0.5 mg testosterone (van Honk and Schutter, 2007), but no differences in emotional recognition accuracy following the nasal administration of 0.9 mg testosterone (van Wingen et al., 2009). In a correlation study on endogenous testosterone levels, Stanton et al. (2009) found no association between testosterone concentrations and amygdala responsiveness to the contrast between angry and neutral faces. Conversely, the three clinical trials included in the review described associations between higher testosterone levels and increased brain activity (Hermans et al., 2008; van Wingen et al., 2009; Bos et al., 2013).

Finally, the oral administration of progesterone (400 mg) did not affect FEP in the only trial comparing this treatment to placebo (van Wingen et al., 2008), although the administration of the hormone was associated with increased bilateral activity in the amygdala.

## Discussion

Taken together, the results of the articles reviewed suggest that hormonal changes mediate the judgment of social stimuli, whether by affecting the accuracy of emotional recognition or the functioning of brain structures implicated in the processing of social stimuli, especially the amygdala. These results were obtained from women in the normal menstrual cycle, users and non-users of oral contraceptives, women during pregnancy and in the postpartum, and clinical trials involving the exogenous administration of hormones.

In the natural menstrual cycle, increased levels of estrogen/estradiol typical of the follicular phase favored the recognition of facial expressions of emotion. This finding lends support to the view that ovarian hormones trigger evolutionary adaptations that are relevant for emotional competence, with the possible purpose of increasing mating chances (Derntl et al., 2008a; Kamboj et al., 2015).

The findings also support the proposition of Macrae et al. (2002) according to which FEP is a sexually dimorphic ability, possibly mediated by sex hormones and especially estrogen/estradiol, since receptors for this hormone are found in several brain areas associated with emotional processing (amygdala, hippocampus, and corpus callosum–Fitch and Denenberg, 1998; Osterlund and Hurd, 2001). In the same direction, Sanders et al. (2002) suggested that cognitive tasks in which women tend to perform better than men, such as FEP, are better performed during periods of increased estrogen levels and vice-versa.

The neuroimaging findings in women in their natural menstrual cycle confirm that the amygdala is a key structure in emotional processing and, more importantly, that its activity is influenced by the concentrations of ovarian hormones along the menstrual cycle. However, evidence on the direction of that influence is controversial. Derntl et al. (2008b) found that progesterone decreases typical of the follicular phase were associated with increased neural activity, which suggests that networks implicated in emotional processing are more excitable in the preovulatory phase, which would favor socioemotional behavior and, thus, mating. In opposition, Gingnell et al. (2012) described increased activity in the left amygdala during the luteal phase compared to the follicular phase, which suggests that progesterone may increase the responsiveness of the amygdala in the face of emotional stimuli, mainly those with negative valence. This view is further supported by the results of van Wingen et al. (2008), which show that the acute administration of progesterone increased amygdala responsiveness to displays of anger and fear.

The results of Gingnell et al. (2012) and van Wingen et al. (2008) are in line with available evidence from studies that assessed the responsiveness of brain structures to the presentation of other emotional stimuli that not facial expressions of emotion (Abler et al., 2013; Bayer et al., 2014) and point to an inhibitory influence of estrogen/estradiol upon different networks, while progesterone seems to increase neural responses, especially in the presence of negative emotions (Goldstein et al., 2005; Andreano and Cahill, 2010; Ossewarde et al., 2010). In our review, the levels of estrogen/estradiol were not associated with any specific pattern of activation of the brain structures investigated.

The results of the correlation analyses showed that increased progesterone levels were associated with improved recognition of fearful and disgusted expressions and increased response bias for angry expressions. These findings lend support to previous observations that progesterone is an anxiogenic agent (Akwa et al., 1999; Hiroi and Neumaier, 2006; Derntl et al., 2008a), favoring greater sensitivity or hypervigilance to threatening and contagious faces. According to Conway et al. (2007), increased concentrations of progesterone, commonly observed in the preparation of the organism for pregnancy, would favor adaptive psychological changes that could aid women to face challenges during pregnancy; for example, by improving the recognition of contamination sources that are harmful to mother and baby so as to mitigate external hazards that could affect fetal development. These views are further supported by evidence showing that higher concentrations of progesterone were associated with increased repulse to facial signs and potential sources of disease, such as paleness (Jones et al., 2005; Fleischman and Fessler, 2011), and to possible sources of contamination in food preferences during pregnancy (Flaxman and Sherman, 2000; Fessler, 2002; Fessler et al., 2005).

Conversely, the greater sensitivity to stimuli depicting anger associated with increased progesterone and decreased estrogen/estradiol levels could lead to negative mood (Derntl et al., 2008a) and could be associated with the etiology of premenstrual tension. These hypotheses are supported mainly by the fact that progesterone and estrogen/estradiol have significant modulatory effects on neurotransmitters involved in the regulation of affect and behavior, such as noradrenaline and serotonin (Bethea et al., 1998; Epperson et al., 1999; Amin et al., 2005; Derntl et al., 2008a; Sabino et al., 2016), which are also implicated in depression.

The studies involving users of oral contraceptives also described alterations in FEP, in consonance with previous evidence of contraceptive-related changes in emotional memory, decision-making, face preference, jealousy levels and others (Hamstra et al., 2015). Specifically, in studies about FEP, the use of oral contraceptives was associated with reduced accuracy in the recognition of negative facial expressions. These results provide a basis for the interpretation of previous findings regarding the efficacy of oral contraceptives in the treatment of mood symptoms associated with premenstrual dysphoric disorder, as it points to a possible mechanism of action linked to the reduction in the sensitivity to negative emotions that could underlie the therapeutic effects described in the literature (Freeman et al., 2001; Yonkers et al., 2005).

On the other hand, the use of oral contraceptives was also associated with reduced activation of brain regions implicated in different social functions and in the response to positive emotional stimuli, pointing to possible adverse effects of contraceptives. This finding highlights the role of sex hormones in the facilitation of social affiliation and self-protection.

Regarding the recognition of facial emotions by pregnant women, the increased accuracy in the detection of negative emotions during pregnancy could be explained by the influence of estrogen/estradiol in the amygdala, in line with the results of Pearson and Lewis (2005) and Derntl et al. (2008a) and with evolutionary theories, where the hypervigilance to signs of threat would be a selective advantage for women, especially those about to become mothers.

To Roos et al. (2011), the activation of brain areas during the display of fearful faces could be associated with the levels of testosterone during pregnancy. According to this view, pregnancy would be associated with an increase in the response to threat as an adaptive function of the species.

Considering the reduced number of studies in the pregnancy/postpartum group, these findings should be interpreted with caution as they are speculative and still require replication.

The results of the only study involving women in the postpartum included in this review (Gingnell et al., 2015) showed increased activation of the insula, inferior frontal gyrus and middle frontal gyrus during this period. The authors speculated that, if on the one hand this increased responsiveness could be associated with increased vulnerability to depressive and anxious conditions in the postpartum, on the other hand it would favor effective parenting.

The results of studies on the exogenous administration of testosterone in women showed that it enhances the activation and connectivity between brain structures involved not only in aggressive responses (Hermans et al., 2008), but also in the processing of different socially relevant stimuli. This effect seems to be independent of the affective valence of the stimuli, as suggested by evidence that the basolateral and superficial amygdala had equally increased activation associated with the administration of testosterone during the processing of fear or happiness (Bos et al., 2013).

In conclusion, sex hormones have a significant impact on FEP in women that seem to have an adaptive role, whether related to mating, reproduction, or offspring care. Conversely, these hormones also seem to have a negative impact on mood symptoms associated with premenstrual tension.

The findings described show that the hormonal condition of women is an important variable to be considered in clinical studies involving FEP, as it may act as a confounding variable and favor the occurrence of biases. To our knowledge, this type of methodological control has often been neglected in studies in the area.

Among the limitations of the studies reviewed here, we should mention the lack of standardized procedures to assess FEP, which frequently hinders specific comparisons, and the lack of consensus about the determination of the different phases of the menstrual cycle, added to the fact that some studies failed to measure/inform hormone concentrations in their subjects, which would be ideal for the establishment of these parameters. Finally, the studies included in the review involved mainly young adult women, leaving a gap of data concerning pregnant women and women in the postpartum, puberty, and pre- or post-menopause, which should be the focus of future investigations.

## Author contributions

FdLO, JdPC, RM-S, JMdS, OP-N conception/design of the work; FdLO, JdPC, RM-S acquisition and analysis of data for the work; FdLO, JdPC, RM-S, JMdS, OP-N interpretation of data for the work; FdLO, JdPC, JPMS draft the work; FdLO, JMdS, OP-N, RM-S review critically for important intellectual content of the work; FdLO, JdPC, RM-S, JMdS, OP-N Final approval of the version to be published; FdLO, JdPC, RM-S JMdS, OP-N Agreement to be accountable for all aspects of the work in ensuring that questions related to the accuracy or integrity of any part of the work are appropriately investigated and resolved.

### Conflict of interest statement

The authors declare that the research was conducted in the absence of any commercial or financial relationships that could be construed as a potential conflict of interest.
