# Supplementary material for: Sex Hormones and Processing of Facial Expressions of Emotion: A Systematic Literature Review
Source: Front Psychol. 2018 Apr 11;9:529. doi: 10.3389/fpsyg.2018.00529 (PMC5904259; doi:10.3389/fpsyg.2018.00529)
Supplement: Supplementary file 1 [file Table1.docx]

**Supplementary Table A –** Description of the procedures used in the facial emotion recognition tasks in the studies reviewed.

| **Study** | **Type of task** | **Stimuli assessed** | **Stimuli**  **set** | **Gender of stimuli** | **Emotions** | **Intensity of**  **emotions** | **Outcome**  **variable** |
| --- | --- | --- | --- | --- | --- | --- | --- |
| ***Menstrual Cycle*** | | | | | | | |
| Pearson  and  Lewis  (19) | Static | 60 | PFAS  Ekman & Friesen (1976) | M/F | A, D, F, H, SA, SU | 100% | Accuracy  Response time |
| Derntl  et al.  (20) | Static | 36 | Gur et al. (2002) | M/F | A, D, F, H, SA + Neutral | 100% | Accuracy  Response bias |
| Derntl  et al.  (21) | Static | 72 | Gur et al. (2002) | M/F | A, D, F, H, SA + Neutral | 100% | Accuracy  Response time  Brain Activation (fMRI) |
| Guapo  et al.  (22) | Dynamic | 240 | PFAS  Ekman & Friesen (1976) | M/F | A, D, F, H, SA, SU + Neutral | Morphing – 10 frames  0 – 100% | Accuracy |
| Derntl et al.  (23) | Static | 36 | Gur et al. (2002) | M/F | A, D, F, H, SA + Neutral | 100% | Accuracy  Response time |
| Kamboj  et al.  (24) | Dynamic | 144 | NimStim  Tottenham et al. (2009) | M/F | A, D, F, H, SA + Neutral | Morphing – 10 frames  0 – 100% | Accuracy  Response bias  Response time |
| Conway  et al.  (25) | Dynamic | 12 | Karolinska Directed Emotional Faces | M/F | D, F, H |  | Accuracy  Intensity |
| Rubinow  et al.  (26) | Static | 40 | Gur et al. (1992) | M/F | H, SA + Neutral | 100% | Response bias |
| Rubin  et al.  (27) | Static | 40 | PEAT  Erwin et al. (1992)  Kohler et al. (2000) | M/F | H, SA + Neutral | 100% | Accuracy  Response time |
| Gingnell  et al.  (28) | Static | 18 | PFAS  Ekman & Friesen (1976) | M/F | A, F | 100% | Accuracy  Response time  Brain Activation (fMRI) |
| Zhang  et al.  (29) | Static | 180 | CFAPS  Wang & Luo (2005) | M/F | A, D, F, H, SA + Neutral | 100% | Accuracy |
| *Oral Contraceptives* | | | | | | | |
| Maner and Miller  (30) | Static | 56 | MacArthur Network Face Stimuli | M/F | A, D, F, AS | 100% | Accuracy |
| Hamstra  et al.  (31) | Dynamic | 204 | PFAS  Ekman & Friesen (1976) | M/F | A, D, F, H, SA + Neutral | Morphing – 10 frames  0 – 100% | Accuracy  Intensity  Response time |
| Radke and  Derntl  (32) | Static | 36 | Gur et al. (2002) | M/F | A, D, F, H, SA + Neutral | 100% | Accuracy |
| Hamstra  et al.  (33) | Dynamic | 204 | PFAS  Ekman & Friesen (1976) | M/F | A, D, F, H, SA | Morphing – 10 frames  0 – 100% | Accuracy |
| Hamstra  et al.  (34) | Dynamic | 204 | PFAS  Ekman & Friesen (1976) | M/F | A, D, F, H, SA | Morphing – 10 frames  0 – 100% | Accuracy  Response time |
| Gingnell  et al.  (35) | Static | 18 | PFAS  Ekman & Friesen (1976) | M/F | A, F | 100% | Accuracy  Response time  Brain Activation (fMRI) |
| Hamstra  et al.  (36) | Dynamic | 204 | PFAS  Ekman & Friesen (1976) | M/F | A, D, F, H, SA + Neutral | Morphing – 10 frames  0 – 100% | Accuracy  Response time |
| *Pregnancy / Postpartum* | | | | | | | |
| Pearson  et al.  (37) | Static | 60 | PFAS  Ekman & Friesen (1976) | M/F | A, D, F, H, SA, SU | 100% | Accuracy |
| Ross  et al.  (38) | Dynamic | 32 | PFAS  Ekman & Friesen (1976) | M/F | A, D, F, H + Neutral | Morphing – 20 frames | Brain Activation (fMRI) |
| Gingnell  et al.  (39) | Static | 24 | PFAS  Ekman & Friesen (1976) | M/F | A, F | 100% | Accuracy  Response time  Brain Activation (fMRI) |
| *Testosterone* | | | | | | | |
| Stanton  et al.  (40) | Static | 20 | NimStim  Tottenham et al. (2009) | M/F | S, R + Neutral | 100% | Brain Activation (fMRI) |
| van Honk and Schutter (41) | Dynamic | NI | Adapted from Montagne et al. (2005) | M/F | A, D, F, H, SA, SU + Neutral | Morphing – 10 frames  0 – 100% | Accuracy  Intensity |
| Hermans  et al.  (42) | Static | 56 | PFAS  Ekman & Friesen (1976)  and  Karolinska Directed Emotional Faces | M/F | A, H | 100% | Brain Activation (fMRI) |
| van Wingen et al.  (43) | Static | 18 | PFAS  Ekman & Friesen (1976) | M/F | A, F | 100% | Accuracy  Response time  Brain Activation (fMRI) |
| Bos  et al.  (44) | Dynamic | 16 | PFAS  Ekman & Friesen (1976) | M/F | F, H | Morphing – 4 frames  55%, 70%, 85%, 100% | Brain Activation (fMRI) |
| *Progesterone* | | | | | | | |
| van Wingen et al.  (45) | Static | 18 | PFAS  Ekman & Friesen (1976) | M/F | A, F | 100% | Accuracy  Response time  Brain Activation (fMRI) |

A = anger; CFAPS = Chinese Facial Affective Picture System; D = disgust; F = fear; fMRI = Functional Magnetic Resonance Imaging; H = happiness; M/F = male/female; NI = not informed; PEAT = Penn Emotion Acuity Test; PFAS = Pictures of Facial Affect; SA = sadness; SU = surprise.
